# Microbiota of Cervical Canal in Nine Patients Diagnosed with Ectopic Pregnancy: Case Series

**DOI:** 10.3390/life15060949

**Published:** 2025-06-12

**Authors:** Kinga Bednarek, Katarzyna Wszołek, Monika Szewc, Mirosława Gałęcka, Adrian Mruczyński, Alan Bruszewski, Marcin Wierzchowski, Maciej Wilczak, Karolina Chmaj-Wierzchowska

**Affiliations:** 1 Department of Maternal and Child Health and Minimally Invasive Surgery, Poznan University of Medical Sciences, 60-535 Poznań, Polandadrianmrucz@gmail.com (A.M.); abruszewski@ump.edu.pl (A.B.); mwil@ump.edu.pl (M.W.); 2 Institute of Microecology, 60-129 Poznań, Poland; monika.szewc@instytut-mikroekologii.pl (M.S.); drgalecka@instytut-mikroekologii.pl (M.G.); 3 Department of Chemical Technology of Drugs, Poznan University of Medical Sciences, 60-535 Poznań, Poland; mwierzch@ump.edu.pl

**Keywords:** microflora, ectopic pregnancy, cervical canal

## Abstract

Dysbiosis, or an altered microbiota composition, has been implicated in chronic endometrial inflammation and recurrent implantation failure. Despite growing research on the relationship between the genital microbiome and reproductive health, few studies have examined its role in ectopic pregnancy. Therefore, our study focuses on the microbiota of the cervical canal in women diagnosed with an ectopic pregnancy. **Material and methods:** The study group consisted of nine women of a reproductive age who were hospitalized at the Department of Maternal and Child Health, Gynecology and Obstetrics, Clinical Hospital of the University of Poznań, between February and September 2023. In nine patients, an ectopic pregnancy was diagnosed based on a transvaginal ultrasound examination. The swabs were collected for quantitative microbiological culture (using Amies transport medium). The microbiological analyses involved quantitative culture on selected selective and differential media, following the Standard Operating Procedure developed by the Institute of Microecology. **Results:** A reduced *Lactobacillus* spp. count (≤5 × 10^7^ CFU/mL) was observed in 78% of the patients participating in the study, including those that produce H_2_O_2_, i.e., with strong protective properties for the environment of the female reproductive tract. The molecular analyses revealed *Ureaplasma* spp. (*U. parvum* and *U. urealyticum*) in 33% of the samples (three patients). However, *Chlamydia trachomatis* and *Mycoplasma genitalium* were not detected in any of the analyzed samples. **Conclusions:** The ease of obtaining material and the minimally invasive nature of lower reproductive tract examinations may allow for the evaluation of microbiota imbalances, helping to identify individuals at an increased risk of reproductive complications.

## 1. Introduction

Ectopic pregnancy is a significant health problem, leading to morbidity and mortality in women. Early diagnosis and treatment remain challenging. It occurs when a fertilized egg implants outside the mucosa of the uterine cavity [1,2]. The vast majority of ectopic pregnancies (97–98%) occur in the fallopian tube [3]. Depending on the clinical situation, three management strategies are used: wait-and-see management, drug treatment with methotrexate (MTX), or surgical intervention via laparotomy or laparoscopy [4,5]. Approximately 33% of women with a diagnosed ectopic pregnancy are qualified for drug treatment. In most of them, there is a chance of successful therapy and avoidance of surgery [1,2,4,6]. The most common risk factors for an ectopic pregnancy include a history of gynecologic surgery, particularly involving the fallopian tubes, infertility treatment with assisted reproductive techniques, and the use of certain contraceptive methods, such as nonhormonal intrauterine devices. A history of pelvic inflammatory disease also increases the risk [2,5]. In the situation of infection with gonorrhea or *Chlamydia trachomatis*, the risk of an ectopic pregnancy increases three times. Damage to the fallopian tubes is one potential mechanism underlying this correlation [7,8,9].

For most women, the vaginal microbiota is a relatively stable and low-diversity ecosystem, comprising approximately 200 bacterial species—9% of the total bacterial population in the human body. Its composition, despite its relatively stable core, differs from woman to woman and is influenced by several factors, including the following: the phase of the menstrual cycle, the hormonal status of the patient—which correlates with the changing age of the woman, the type of contraception used, and the presence of pathological changes. In addition, the composition of the microbiota depends on hygiene and sexual habits. The primary protective bacteria in the female reproductive tract belong to the genus *Lactobacillus*. Five main types of vaginal bacterial communities have been identified: four dominated by *Lactobacillus* species (*L. crispatus*, *L. gasseri*, *L. iners*, and *L. jensenii*) and one characterized by a low percentage or absence of *Lactobacillus* [10,11]. Bacteria of the genus *Lactobacillus* spp. show strong protective properties for the environment of the female reproductive tract. The main defense mechanisms of *Lactobacillus bacilli* include the following: the production of lactic acid, which supports an acidic environment (pH ≤ 4.5) that is unsuitable for pathogenic organisms [12,13], production of bactericidal (bacteriocins) and protective (H_2_O_2_) substances [12,13], barrier functions making it difficult for pathogens to adhere to the vaginal wall [12,13], influence on the speed and efficiency of epithelial renewal, promoting mucus production, making it difficult for pathogens to adhere [12,13], and stimulation of a woman’s immune system by which the microbiota improves her ability to fight off pathogen attacks [12,13]. The cervical microbiota, considered as an SoHO (substance of human origin), plays a key role in women’s gynecological health and, along with the gastrointestinal microflora, can be treated as a therapeutic agent.

An important issue is the effect of the microbiota on fertility and the ability of the endometrium to accommodate the implanting embryo. Dysbiosis, or an altered microbiota composition, has been implicated in chronic endometrial inflammation and recurrent implantation failure. Chronic inflammation, even in the absence of clinical symptoms, is now recognized as a potential cause of infertility [14]. An abnormal microbiota composition in the female genital tract has been linked to pregnancy complications, including implantation failure during assisted reproductive techniques, miscarriage, and preterm delivery. Despite growing research on the relationship between the genital microbiome and reproductive health, few studies have examined its role in ectopic pregnancy. Therefore, our study focuses on the microbiota of the cervical canal in women diagnosed with an ectopic pregnancy.

## 2. Materials and Methods

### 2.1. Materials

#### 2.1.1. Study Group

The study group consisted of 9 women of childbearing age diagnosed with ectopic pregnancy who were hospitalized at the Department of Maternal and Child Health, Gynecological–Obstetrical Clinical Hospital of the University of Poznań from February to September 2023. The eligibility criterion was a diagnosis of ectopic pregnancy based on current clinical guidelines [2]. Microbiological exclusion criteria were as follows: antibiotics or antifungal drugs currently being used and for at least 2 weeks.

After providing informed consent, participants underwent cervical canal swabbing before conservative and/or surgical treatment. The swabs were collected for quantitative microbiological culture and qualitative molecular detection of *Chlamydia trachomatis*, *Mycoplasma genitalium*, and *Ureaplasma* spp.

#### 2.1.2. Chemicals and Media Used

- Amies medium (Deltalab, S.L., Barcelona, Spain) (transport medium for quantitative microbiological culture).
- eNAT^®^ (Copan, Perotti, Italy) (transport medium for qualitative molecular detection of *Chlamydia trachomatis*, *Mycoplasma genitalium*, and *Ureaplasma* spp.).
- AmpliSens^®^ DNA-sorb-AM nucleic acid extraction kit (Ecoli s.r.o, Purkyňova, Slovak Republic) (for bacterial DNA isolation).
- AmpliSens^®^ C.trachomatis/Ureaplasma/M.genitalium-MULTIPRIME-FRT PCR kit (Ecoli Dx, Prague, Czech Republic) (for multiplex real-time PCR).
- Microbiological determinations were conducted using the following media: Transparent Chromogenic UTI Medium (OXOID Deutschland GmbH, Wesel, Germany), Columbia Agar with Sheep Blood (OXOID Deutschland GmbH, Wesel, Germany), Sabouraud Glucose Selective Agar (OXOID Deutschland GmbH, Wesel, Germany), Schaedler Anaerobe KV Selective Agar (OXOID Deutschland GmbH, Wesel, Germany), *Gardnerella* vaginalis selective medium (OXOID Deutschland GmbH, Wesel, Germany), Rogosa + H_2_O_2_ Agar (HEIPHA, Eppelheim, Germany), MacConkey No. 3 (OXOID Deutschland GmbH, Wesel, Germany), Enterococcus Agar (Bile Esculin Azide Agar, GRASO, Krąg, Poland), and Chromogenic *Candida* Selective Agar (GRASO, Krąg, Poland). Identification was based on colony morphology, Gram staining, and biochemical characterization.

### 2.2. Quantitative Microbiological Culture

The swabs were collected for quantitative microbiological culture (using Amies transport medium). Within 48 h of collection, the swabs were delivered to the Microbiology Laboratory of the Institute of Microecology in Poznań (Sielska Street) for analysis. Microbiological analysis involved quantitative culture on selected selective and differential media, following the Standard Operating Procedure developed by the Institute of Microecology. The culture identified bacterial and fungal species (CFU/mL), including *Streptococcus agalactiae* (group B beta-hemolytic streptococcus), *Gardnerella vaginalis*, anaerobic bacteria, other potentially pathogenic bacteria unique to each patient, *Lactobacillus* spp., *Lactobacillus* spp. producing H_2_O_2_, and yeast-like fungi of the genus *Candida*.

### 2.3. Qualitative Detection

The swabs were collected for qualitative molecular detection of *Chlamydia trachomatis*, *Mycoplasma genitalium*, and *Ureaplasma* spp. (using eNAT/(Copan) medium). Qualitative detection of Chlamydia trachomatis, Mycoplasma genitalium, and *Ureaplasma* spp. (*U. parvum* and *U. urealyticum*) involved bacterial DNA isolation from the swabs using AmpliSens^®^ DNA-sorb-AM. The analysis was carried out using multiplex real-time PCR with the AmpliSens^®^ C. trachomatis/Ureaplasma/M. genitalium-MULTIPRIME-FRT PCR kit.

## 3. Results

### 3.1. Study Group Characteristics

The study group included nine women with an ectopic pregnancy, aged 27–42 years, with body mass index (BMI) values ranging from 19.84 to 36.73 kg/m^2^. Obesity (BMI > 30 kg/m^2^) was present in three subjects, and hypothyroidism was diagnosed in two. Of the gynecological surgeries, the patient histories were as follows: two patients had a scraping of the uterine cavity after a miscarriage, a previous ectopic pregnancy was surgically treated in two women, and two had no procedures performed. Only one patient used drugs (letrox and glucophage) during their hospitalization. The initial values for BetaHCG levels ranged from 746.2 to 4422.0 mIU/mL. During hospitalization, nine patients were diagnosed with a tubal ectopic pregnancy. The diagnosis of an ectopic pregnancy was based on a transvaginal ultrasound examination, which identified an ectopic gestation located in the fallopian tube. Methotrexate treatment was administered to nine women; one developed abdominal bleeding after the second dose of MTX with the need for surgical treatment (laparoscopic excision of the right fallopian tube with an ectopic pregnancy). In the remaining clinical situations, the therapeutic stance was wait-and-see. The characteristics of the study group, including age, weight, height, BMI, obstetric history, past surgeries, comorbidities, and initial chorionic gonadotropin value are shown in Table 1a. The clinical diagnosis and type of treatment used are shown in Table 1b.

### 3.2. Qualitative and Quantitative Assessment

In the present study, a decreased number of *Lactobacillus* spp., with strong protective properties for the environment of the female reproductive tract was observed in more than 78% of the patients. A decrease in *Lactobacillus* spp. is most often associated with an imbalance of the ecosystem of the reproductive tract. *Streptococcus agalactiae* colonization was found in one patient, representing 11% of all women in the study. *Gardnerella vaginalis*, one of the bacteria most often responsible for bacterial vaginosis of the vagina, and anaerobic bacteria (no identification; Gram-negative bacilli) were not isolated at all.

Yeast-like fungi were detected in 22% of the patients. *Candida albicans* was identified in the case of one patient, while *Saccharomyces cerevisiae* was found in the case of one patient. Among other bacteria, *Enterococcus* spp. was the most frequently detected (55% of the swabs), followed by *Escherichia coli* (11%) and coagulase-negative *Staphylococcus* spp. (22%). *Staphylococcus aureus* was found in one swab, which accounted for 11% of all tested samples from female patients. The situation was similar for the glucose-unfermented bacillus *Acinetobacter calcoaceticus*, which, according to the literature, may be a natural component of the human microbiota.

The molecular analysis revealed *Ureaplasma* spp. (*U. parvum* and *U. urealyticum*) in 33% of the samples (three patients). However, *Chlamydia trachomatis* and *Mycoplasma genitalium* were not detected in any of the analyzed samples. Table 2 presents the individual results of the quantitative microbiological cultures and qualitative molecular determinations for *C. trachomatis*, *M. genitalium*, and *Ureaplasma* spp. (*U. parvum* and *U. urealyticum*).

## 4. Discussion

The vaginal and cervical environments are populated by aerobic and facultatively anaerobic microorganisms, which exhibit both protective and pathogenic potential [15,16]. Defining the precise boundary between physiological and pathological microbiota compositions remains challenging. Colonization of the reproductive tract by nonprotective microorganisms can occur in both symptomatic and asymptomatic women. Given the incomplete understanding of this area, some researchers suggest that the presence of bacteria in the vagina and cervical canal does not necessarily require treatment unless it causes inflammation or clinical symptoms [17]. However, this does not apply to bacteria with well-established pathogenicity.

Macura et al. [14] indicated the presence of bacteria throughout the female reproductive tract. These bacteria belong to different genera, and the proportion and percentage of each species varies within different parts of the reproductive system. Previously, the fallopian tubes and endometrium were considered sterile sites protected by cervical mucus, which acts as a barrier to bacterial entry into the uterus [18]. However, this view has been challenged in subsequent studies. The bacterial ecosystem of the vagina, despite having a fairly stable core, varies from woman to woman and is influenced by several factors. These include the phase of the monthly cycle, the hormonal status of the patient—correlating with the changing age of the woman (from puberty to a postmenopausal age)—medications taken, fertility, the type of contraception used, and the presence of pathological changes such as endometriosis or benign tumors. In addition, the composition of the microbiota is influenced by hygiene and sexual habits. The source of bacteria in the genital tract can be semen. Some authors point out that semen and the vaginal microbiota complement each other. Bacteria from semen can change the composition of the vaginal microbiota and its pH and reach the uterine cavity, colonizing the endometrium [19].

Despite the growing number of studies on the relationship between the genital microbiome and women’s reproductive health, few have focused on its impact on ectopic pregnancies. One of the risk factors for abnormally located pregnancies is pelvic inflammatory disease, with a threefold increase in risk when infected with *Chlamydia trachomatis*, among others. In the pilot study conducted, *C. trachomatis* and *Mycoplasma genitalium* were not found in the cervical canal. *Ureaplasma* spp. was present in fewer than 33% of the patients, but the literature data do not indicate a higher prevalence of infection with this atypical bacterium in patients with ectopic pregnancies compared to control groups [20].

It should be emphasized, however, that the cervical canal microflora shows high inter-individual variability, which constitutes a significant limitation in the interpretation of the results. Further studies should include an analysis of inter-group variability to allow for more accurate inferences and an assessment of the significance of the observed differences.

During an uncomplicated pregnancy, the vaginal microflora remains stable, with a predominance of *Lactobacillus* species [21]. Other bacteria that may be present in the vaginal microbiome include *Gardnerella vaginalis* (*G. vaginalis*), *Atopobium vaginae* (*At. vaginae*), *Prevotella* spp., *Sneathia amnii* (*S. amnii*), and *Candidatus Lachnocurva vaginae* (*C. Lachnocurva vaginae*) [22]. In addition, the microbiome of the vagina and cervix, where *Lactobacillus* predominates, differs from that of the fallopian tubes. There is a probable relationship between the particularities of cervical dysbiosis and tubal injury, with a secondary increased risk of ectopic pregnancy. Comparing the vaginal microbiome of women with ectopic pregnancies in the fallopian tube versus intrauterine pregnancies in the first trimester, a significantly increased relative abundance of *Gardnerella*, *Prevotella*, *Clostridia*, and *Leptotrichiaceae* was observed. In contrast, no significant changes were found in the relative abundance of *Lactobacillus* [23]. In the present study, a decreased number of *Lactobacillus* spp. was observed in more than 78% of the patients. A decrease *Lactobacillus* spp. is most often associated with an imbalance of the ecosystem of the reproductive tract, which can lead to conditions or complications in the course of pregnancy [24]. A higher percentage of associated microbiota, such as *Enterococcus* spp. or *E. coli*, was also observed in the studied patients compared to the microbiota of the cervical canal of women in the first weeks of pregnancy [16]. In contrast, a study by Ruan et al. [23] demonstrated a significantly higher abundance of *Gardnerella* and *Prevotella* and a lower abundance of *Lactobacillus* in the vaginal microbiome of patients diagnosed with an ectopic fallopian tube pregnancy [23]. In the present study, *G. vaginalis* was not found in the patients. These findings suggest that analyses of the microbiome of the reproductive tract may not only help identify women at a high risk of obstetric complications but also support the development of preventive strategies [25]. Of course, the primary limitation of our study is the small sample size and the lack of a control group. However, as a pilot study, it provides preliminary data that can help refine future research directions.

An important issue is the impact of the microbiota on fertility and the ability of the endometrium to accommodate the implanting embryo. Abnormalities in the composition of the female genital tract microbiota have been associated with various pregnancy complications, including implantation failure in assisted reproductive techniques, miscarriage, and preterm labor. In recent years, increasing attention has been given to chronic endometrial inflammation due to its strong association with impaired fertility. This condition is believed to interfere with key physiological processes, such as oocyte fertilization and embryo implantation, potentially leading to early pregnancy loss [26,27]. Repeated pregnancy loss has also been associated with chronic endometritis [28]. The leading cause of chronic endometritis is infection of the endometrium by microorganisms commonly residing in the lower genital tract. The rationale for studying the correlation between the lower genital tract microbiome and ectopic pregnancy is the practical advantage of easily obtaining samples from the lower genital tract. This is particularly important if identifying a potential risk factor could lead to its use as a screening or diagnostic biomarker for reproductive conditions.

This study provides preliminary insights into the cervical canal microbiota composition in patients diagnosed with an ectopic pregnancy. The main limitations of this work include the small sample size, lack of a control group, and high inter-individual variability in the microbiota composition. Taken together, these factors limit the generalizability and statistical significance of the findings. Nevertheless, the study highlights the potential of lower reproductive tract microbiota analysis as a supportive tool for identifying women at an increased risk of obstetric complications. The ease of sample collection and the minimally invasive nature of the procedure make this approach promising for screening and diagnostic applications.

## 5. Conclusions

The observed alterations in the cervical microbiota—particularly the reduced presence of *Lactobacillus* spp. and increased abundance of potentially pathogenic microorganisms—may suggest an association between microbial dysbiosis and abnormal embryo implantation. These findings underscore the need for further investigation into the role of the lower genital tract microbiome in early pregnancy outcomes.

Future studies should adopt a comparative case–control design involving larger cohorts, including women in early, normal intrauterine pregnancies as a control group. Such studies may facilitate the identification of specific microbial patterns or taxa that could serve as biomarkers for ectopic pregnancy risk.

## Figures and Tables

**Table 1:** Characteristics of nine women of childbearing age diagnosed with ectopic pregnancy who were hospitalized at the Department of Maternal and Child Health, Gynecological–Obstetrical Clinical Hospital of the University of Poznań from February to September 2023 (**a.** age, weight, height, BMI, obstetric history, past surgeries, comorbidities, and initial BHCG value; **b.** diagnosis and treatment).

**(a)**										
**Patient**	**Age**	**Obstetric History**	**Body Weight [kg]**	**Height [cm]**	**BMI**	**Recent Cytology Result**	**Additional Diseases**	**Past Surgeries**	**Drugs Used**	**Initial BHCG Value [mIU/mL]**
1	36	(1-0-4)	92	163	34.63	Not provided	Obesity	2019—laparoscopy, excision of right fallopian tube, ectopic pregnancy	Lack	746.2
2	36	(0-0-2)	58	164	21.56	Not provided	Lack	2021—ectopic pregnancy; 2021—hydrops of fallopian tubes, bilateral removal	Lack	2727
3	27	(0-0-2)	119	180	36.73	Not provided	Obesity	2022—abrasion after miscarriage	Lack	3258.0
4	42	(0-0-1)	70	171	23.94	Not provided	Hypothyroidism, insulin resistance	Not provided	levothyroxine (100 µg) and metformin (1000 mg XR)	914.0
5	35	(1-0-0)	94	168	33.3	Not provided	Obesity	2022—abrasion after miscarriage at 16 wp (triploidy)	Lack	4422.0
6	30	(0-0-0)	56	168	19.84	Not provided	Lack	Hysteroscopy—removal of endometrial polyp	Lack	2493.0
7	30	(0-0-0)	54	163	20.32	2023—result normal	Lack	Cesarean section	Lack	1604.0
8	43	(2-0-0)	63	168	22.32	2023—result normal	Hashimoto’s disease	Not provided	Lack	4136.0
9	41	(0-0-2)	56	161	21.6	2023—result normal	Lack	2010—laparoscopic cholecystectomy	Lack	991.2
**(b)**										
**Patient**	**Diagnosis**							**Treatment**		
1	Fallopian tube pregnancy on the left side—leprous miscarriage. Status after removal of the right fallopian tube due to ectopic pregnancy. Obesity							Observation. Leprous miscarriage		
2	Ectopic pregnancy of unknown location—pregnancy in the uterine part of the fallopian tube. Obstructive obstetric history. Status after left ectopic fallopian tube pregnancy (2021). Status after laparoscopic removal of both fallopian tubes (2021). Condition after infertility treatment							Two doses of Methotrexate 100 mg IM		
3	Pregnancy III, week 4, left fallopian tube pregnancy. Obesity							One dose of MTX 100 mg IM		
4	Pregnancy II, week 7. Left-sided ectopic fallopian tube pregnancy. Bleeding from the genital tract. Hypothyroidism. Insulin resistance. Status after removal of endometrial polyp							Two doses of MTX 100 mg IM		
5	Pregnancy II, week 10, ectopic right fallopian tube pregnancy. Condition after cesarean section							Two doses of MTX 100 mg IM		
6	Right-sided ectopic fallopian tube pregnancy							Two doses of MTX 100 mg IM		
7	Right-sided fallopian tube pregnancy. Unicornuate uterus—no left adnexa. Post-hemorrhagic anemia							Two doses of MTX 100 mg IM, then bleeding into peritoneal cavity. Laparoscopic excision of right fallopian tube		
8	Pregnancy III, week 6. Right-sided ectopic fallopian tube pregnancy							Two doses of MTX 100 mg IM		
9	Right-sided ectopic fallopian tube pregnancy							Two doses of MTX 100 mg IM		

**Table 2:** Detailed results of protective, associated, and pathogenic microflora of quantitative microbiological cultures and qualitative determination by molecular method for *Chlamydia trachomatis*, *Mycoplasma genitalium*, and *Ureaplasma* spp. (*U. parvum* and *U. urealyticum*).

**Microflora**		**Patient 1.**	**Patient 2.**	**Patient 3.**	**Patient 4.**	**Patient 5.**
protective	*Lactobacillus* spp. H_2_O_2_ RV ≥ 5 × 10^7^, S = 2 × 10^5^	9 × 10^7^	5 × 10^8^	↓ RV	6 × 10^7^	4 × 10^5^
associated	*Lactobacillus* spp. RV ≥ 5 × 10^7^, S = 2 × 10^5^	9 × 10^7^	5 × 10^8^	↓ RV	6 × 10^7^	4 × 10^5^
associated	*Streptococcus* spp. RV < 2 × 10^4^	↓ RV	↓ RV	*S. agalactiae* 4 × 10^4^	↓ RV	↓ RV
associated	Anaerobic bacteria RV < 2 × 10^6^	↓ RV	↓ RV	↓ RV	↓ RV	↓ RV
associated	*Gardnerella vaginalis* RV < 2 × 10^4^	↓ RV	↓ RV	↓ RV	↓ RV	↓ RV
associated	*Candida* spp.and other yeast-like fungi RV < 2 × 10^4^	↓ RV	*Saccharomyces cerevisiae* 6 × 10^6^	*Candida albicans* 2 × 10^5^	↓ RV	↓ RV
associated	*Enterococcus* spp. RV < 2 × 10^4^	3 × 10^5^	8 × 10^5^	4 × 10^4^	↓ RV	↓ RV
associated	*E. coli* RV < 2 × 10^4^	4 × 10^5^	↓ RV	↓ RV	↓ RV	↓ RV
pathogenic	*Staphylococcus aureus* RV < 2 × 10^4^	↓ RV	3 × 10^5^	↓ RV	↓ RV	↓ RV
associated	*Staphylococcus* spp. *CNS* RV < 2 × 10^4^	↓ RV	↓ RV	↓ RV	↓ RV	1 × 10^5^
pathogenic	*Acinetobacter calcoaceticus* RV < 2 × 10^4^	↓ RV	↓ RV	↓ RV	↓ RV	↓ RV
pathogenic	*Chlamydia trachomatis*	(−)	(−)	(−)	(−)	(−)
pathogenic	*Ureaplasma* spp.	(−)	(−)	(+)	(−)	(−)
pathogenic	*Mycoplasma genitalium*	(−)	(−)	(−)	(−)	(−)
**Microflora**		**Patient 6.**	**Patient 7.**	**Patient 8.**	**Patient 9.**	
protective	*Lactobacillus* spp. H_2_O_2_ RV ≥ 5 × 10^7^, S = 2 × 10^5^	1 × 10^9^	7 × 10^8^	4 × 10^7^	↓ RV	
associated	*Lactobacillus* spp. RV ≥ 5 × 10^7^, S = 2 × 10^5^	1 × 10^9^	7 × 10^8^	4 × 10^7^	↓ RV	
associated	*Streptococcus* spp. RV < 2 × 10^4^	↓ RV	↓ RV	↓ RV	↓ RV	
associated	Anaerobic bacteria RV < 2 × 10^6^	↓ RV	↓ RV	↓ RV	↓ RV	
associated	*Gardnerella vaginalis* RV < 2 × 10^4^	↓ RV	↓ RV	↓ RV	↓ RV	
associated	*Candida* spp. and other yeast-like fungi RV < 2 × 10^4^	↓ RV	↓ RV	↓ RV	↓ RV	
associated	*Enterococcus* spp. RV < 2 × 10^4^	1 × 10^4^	↓ RV	↓ RV	4 × 10^7^	
associated	*E. coli* RV < 2 × 10^4^	↓ RV	↓ RV	↓ RV	↓ RV	
pathogenic	*Staphylococcus aureus* RV < 2 × 10^4^	↓ RV	↓ RV	↓ RV	↓ RV	
associated	*Staphylococcus* spp. *CNS* RV < 2 × 10^4^	↓ RV	↓ RV	↓ RV	7 × 10^5^	
pathogenic	*Acinetobacter calcoaceticus* RV < 2 × 10^4^	2 × 10^4^	↓ RV	↓ RV	↓ RV	
pathogenic	*Chlamydia trachomatis*	(−)	(−)	(−)	(−)	
pathogenic	*Ureaplasma* spp.	(+)	(−)	(+)	(−)	
pathogenic	*Mycoplasma genitalium*	(−)	(−)	(−)	(−)	

S—standard, RV—reference value, ↓ RV—below reference value, (+)—positive, and (−)—negative.

## Data Availability

The data presented in this study are available on request from the corresponding author.
